# Synergistic Enhancement of Phenolic Profile, Color Stability, and Aroma Complexity in Marselan Wine Through Co-Fermentation with Fu Tea

**DOI:** 10.3390/foods15152652

**Published:** 2026-07-28

**Authors:** Dolkar, Hexiang Bai, Ziyu Wang, Jihong Yang

**Affiliations:** 1 College of Food Science and Pharmacy, Xinjiang Agricultural University, Urumqi 830000, China; zhuoga20000129@outlook.com (D.); 15531946134@163.com (H.B.); s3siwzy@163.com (Z.W.); 2 College of Enology, Northwest A&F University, Yangling 712100, China

**Keywords:** tea wine, Marselan grape, Fu brick tea, phenolic compounds, copigmentation, aroma synergy, co-fermentation

## Abstract

Co-fermentation of tea and wine offers a promising route to functional beverages with enhanced sensory complexity and potential health benefits. We co-fermented Marselan (*Vitis vinifera* L.) grapes with Fu brick tea (0, 0.5, 1.0, 1.5 g/L), a post-fermented tea containing the probiotic fungus *Eurotium cristatum*. We systematically examined how tea-derived phenolics modulate wine color and aroma. Fu tea addition progressively decreased alcohol (12.24% → 10.86%) and modestly reduced residual sugar, while volatile acidity remained stable, indicating no adverse fermentation impact. HPLC revealed a substantial increase in tea-derived phenolics; chlorogenic acid nearly doubled and became the dominant phenolic compound. CIELab color analysis showed increased lightness (L) and marked decreases in yellow hue (b) and hue angle (h°). Total phenolic content correlated negatively with h° (r = −0.656, *p* = 0.0205), suggesting that the overall phenolic load—not any single compound—is associated with the shift toward a more stable brick-red hue. PCA showed that chlorogenic acid contributed to both color (PC1) and phenolic (PC2) variation. GC-MS revealed substantial increases in esters and higher alcohols, and the emergence of 1-octen-3-ol, a characteristic metabolite of *E. cristatum*. C6 compounds associated with herbaceous aromas decreased significantly. Fu tea co-fermentation thus enhanced wine color stability and aroma complexity through a multifaceted phenolic network. Under our conditions, 1.0 g/L Fu tea gave the most favorable balance of color stability and aroma complexity. These findings provide a preliminary theoretical basis for developing tea-wine products, though further mechanistic studies are needed to establish causality.

## 1. Introduction

Consumers increasingly demand innovative beverages that offer both unique sensory experiences and potential health benefits. Tea wine is a fermented alcoholic beverage produced by co-fermenting tea leaves or tea extracts with sugar sources (e.g., grape must, honey, or cereals). Tea wine—combining refined tea flavors with fermented wine complexity—represents a compelling frontier in functional beverage development. Marselan (*Vitis vinifera* L. cv. Marselan, VIVC number 16383) [1] is a cross between Cabernet Sauvignon and Grenache. It was bred by Paul Truel in 1961 at INRA (Institut National de la Recherche Agronomique), Montpellier, France (breeding number INRA 1810-68), and officially introduced into China in 2001 [1]. Marselan is a mid–late maturing red grape variety. It has emerged as a promising grape variety in China’s key wine regions, owing to its deep color, robust tannins, and distinctive black-fruit and spice aromas [2,3]. Currently, Marselan is cultivated on over 60,000 mu (~4000 hectares) across China, with the eastern foothills of the Helan Mountains in Ningxia accounting for nearly 65% of the total area, followed by Xinjiang (>5000 mu), Hebei, and Shanxi [4].

The Yanqi Basin in Xinjiang—where the grapes for this study were sourced—is characterized by gravelly sandy soils, a continental arid climate, and large diurnal temperature variations, which favor the accumulation of anthocyanins, polyphenols, and aroma precursors in the grapes. However, monomeric anthocyanins are inherently unstable, and aroma compounds are susceptible to oxidation during vinification and aging. These challenges limit the shelf-life and organoleptic quality of Marselan wines [5]. Innovative vinification techniques that enhance color stability and enrich aromatic complexity are therefore of considerable interest.

Fu brick tea is a uniquely Chinese post-fermented dark tea. It features a proprietary “fungal fermentation” stage that promotes *Eurotium cristatum* growth [6]. This “golden flower” fungus produces the tea’s characteristic fungal, woody, and mushroom-like aroma. It also synthesizes bioactive secondary metabolites: phenolic acids, flavonoids, and polysaccharides [7]. These compounds confer antioxidant and potential probiotic properties to Fu tea extracts [8]. Phenolic compounds are key quality determinants in both wine and tea, influencing color, astringency, mouthfeel, and antioxidant activity. In wine, anthocyanin stability is largely governed by copigmentation—non-covalent interactions between anthocyanins and colorless phenolic cofactors that protect the chromophore from nucleophilic attack and enhance color intensity and stability [9]. Adding plant-based adjuncts—grape stems or tea leaves—during fermentation can modulate wine’s phenolic matrix. This improves color stability through intermolecular copigmentation and shapes aroma profiles via synergistic or antagonistic interactions [10,11]. Tea polyphenols—especially flavan-3-ols like catechins—can engage in π–π stacking or polymerize with grape-derived anthocyanins. These interactions form more stable pigment complexes and enhance color stability [12]. *E. cristatum*’s unique metabolites may also interact with yeast-derived fermentation volatiles, creating a more complex and distinctive flavor matrix [13].

Despite this potential, no study has systematically investigated the co-fermentation of Marselan grapes with Fu tea. We hypothesized that Fu tea co-fermentation would enrich tea-derived phenolics, improve color stability via copigmentation, and introduce characteristic fungal aromas. This study aims to construct a Marselan-Fu tea co-fermentation system to answer three questions: (1) How does Fu tea addition affect phenolic profile and color? (2) What role do tea-derived phenolics play in coordinating wine color? (3) How does co-fermentation reshape the volatile aroma profile? Our findings aim to provide a mechanistic basis for developing premium, differentiated tea-wine products, and to support high-value utilization of Fu tea resources. This study is intended for researchers and practitioners in the wine and functional beverage industries, and the results may inform the development of commercial tea-wine products with improved color stability and aroma complexity.

## 2. Materials and Methods

### 2.1. Materials and Reagents

Marselan grapes utilized in this study were harvested from the Guofei Vineyard, Qigexing Town, Yanqi Hui Autonomous County, Bayingolin Prefecture, Xinjiang, China (approximately 42° N, 86° E). The region has a continental arid climate with low annual precipitation (50–80 mm), abundant sunshine (>3000 h per year), and a large diurnal temperature variation, which favors the accumulation of phenolic compounds and anthocyanins in the grapes. The vineyard has gravelly sandy soils and is managed under organic practices with minimal pesticide use.

The Fu brick tea used in this study was procured from the Xi’an tea market. It originates from Jingyang County, Shaanxi Province, China—a region recognized for its traditional production of Fu brick tea as a national geographical indication product. The tea is produced from Shaanxi dark raw tea through a proprietary solid-state fermentation (“fungal flowering”) process involving *Eurotium cristatum*, which is responsible for its characteristic fungal and woody aroma. The tea was stored in sealed containers at room temperature away from light prior to use. A brief characterization of the tea lot used in this study revealed a moisture content of 7.2% and a total phenolic content of 18.5 mg GAE/g. The tea exhibited abundant “golden flowers” (*E. cristatum* colonies) and a clean, typical fungal-floral aroma. The appearance of the Fu brick tea and its characteristic “golden flowers” are shown in Figure S7 (Supplementary Materials). Four treatment groups corresponding to distinct Fu tea addition levels were established for the production of Fu tea–Marselan wine.

Analytical grade reagents, including hydrochloric acid, Folin–Ciocalteu reagent, anhydrous sodium carbonate, tannic acid, and Folin–Denis reagent, were sourced from Xi’an Chemical Reagent Factory. Malvidin-3-O-glucoside standard (≥98% purity) was obtained from Sigma-Aldrich (St. Louis, MO, USA). Tartaric acid (analytical grade) was purchased from Sinopharm Chemical Reagent Co., Ltd. (Shanghai, China). Chromatographic grade solvents, comprising methanol, acetonitrile, ethyl acetate, isopropanol, formic acid, and acetic acid, were acquired from Xi’an Chemical Reagent Factory.

Total phenols were determined using the Folin–Ciocalteu method [14], with gallic acid as the standard, and expressed as mg GAE/L. Total tannins were measured by the Folin-Denis method [15], using tannic acid as the standard. Total anthocyanins were quantified by the pH-differential method [14] and expressed as mg malvidin-3-O-glucoside equivalents per liter.

### 2.2. Instruments and Equipment

Key instrumentation and apparatus employed included: a gas chromatograph–mass spectrometer (Trace 1610-ISQ, Thermo Fisher Scientific, Waltham, MA, USA); a wine color analyzer (W100, Hanon Advanced Technology Group Co., Ltd., Jinan, China); a high-performance liquid chromatography system equipped with a ShimNex CS C18 column (Shimadzu (Shanghai) Experimental Equipment Co., Ltd., Shanghai, China); a FOSS WineScan™ SO_2_ automatic wine analyzer (FOSS Analytical, Hillerød, Denmark); an electronic analytical balance (LE204E/02, Mettler-Toledo Instruments (Shanghai) Co., Ltd., Shanghai, China); and a thermostatic electric water bath (HWS-26, Shanghai Yiheng Scientific Instruments Co., Ltd., Shanghai, China).

### 2.3. Experimental Methods

#### 2.3.1. Preparation of Fu Tea–Marselan Wine

Premium Marselan grapes were subjected to manual sorting, after which 30% of the fruit mass underwent gentle destemming and crushing, followed by alcoholic fermentation with skin contact (maceration-fermentation). The crushed must was filled into 5 L glass carboys at approximately 70% capacity. Potassium metabisulfite (30 mg/L) was added to the must prior to fermentation. The resulting must was inoculated with 0.2 g/L of FX15 *Saccharomyces cerevisiae* active dry yeast (Angel Yeast Co., Ltd., Yichang, China). The Fu tea leaves were ground into a fine powder (passing through a 40-mesh sieve) using a laboratory grinder and added directly to the must without further treatment at mass concentrations of 0 (control), 0.5, 1.0, and 1.5 g/L, respectively, for co-fermentation with the yeast inoculum. Alcoholic fermentation was conducted under controlled temperature conditions ranging from 25 ± 2 °C and was allowed to proceed for 12–14 days until residual sugar content stabilized. Punching down (pigeage) was performed daily during fermentation. Upon completion of alcoholic fermentation, the pomace was separated by pressing, and the wine was racked. The resultant young wine was subsequently aged at 20 °C for a period of one month. Following aging, the wine was clarified by vacuum filtration using 0.45 μm aqueous membrane filters (LABFILTER) and a vacuum filtration apparatus (Tianjin Hengao Technology Development Co., Ltd., Tianjin, China), and the finished wine samples were stored under refrigerated conditions at 4 °C in the dark for subsequent analysis.

#### 2.3.2. Determination of Basic Physicochemical Indices

Basic physicochemical parameters of Fu tea–Marselan wine samples across different tea addition levels, including alcohol content (% *v*/*v*), volatile acidity (g/L), residual sugar (g/L), pH, total acidity (g/L), and glycerol content (g/L), were determined using a FOSS WineScan™ Flex automatic wine analyzer (Type 79063, FOSS Analytical, Hillerød, Denmark). The instrument was calibrated with a reference wine of known composition, and the measurements were performed according to the manufacturer’s protocols.

#### 2.3.3. Colorimetric Analysis

Wine samples were filtered through 0.45 μm membrane filters prior to analysis, with measurements conducted at ambient temperature (20–25 °C). The W100 color analyzer was preheated for 30 min. Prepared samples were transferred to dedicated cuvettes, positioned within the instrument’s measurement chamber, and the cover was closed. CIELab color space parameters (L*, a*, b*, C*, h°) were acquired using the accompanying instrument software.

#### 2.3.4. Analysis of Volatile Aroma Compounds

An aliquot of 5 mL of wine sample and 1 g of NaCl were placed into a 10 mL headspace vial, followed by the addition of 10 μL of 4-methyl-2-pentanol (100 mg/L in ethanol) as an internal standard and vortex mixing. The HS-SPME conditions were: incubation at 40 °C for 30 min with agitation, extraction with a 50/30 μm DVB/CAR/PDMS fiber (Supelco, Bellefonte, PA, USA), and desorption at 250 °C for 5 min. GC–MS analysis was performed on a DB-Wax column (30 m × 0.25 mm × 0.25 μm film thickness, Agilent Technologies, Santa Clara, CA, USA) with helium as the carrier gas at a constant flow rate of 1.0 mL/min. The oven temperature program was as follows: initial temperature 40 °C held for 3 min, then ramped to 230 °C at 4 °C/min and held for 10 min. The injector temperature was maintained at 250 °C, and the injection was performed in splitless mode. The ion source temperature was set at 230 °C, the quadrupole temperature at 150 °C, and the transfer line at 250 °C. Mass spectra were acquired in full scan mode over the *m*/*z* range 35–350. Compound identification was based on comparison of mass spectra with the NIST 17 library and retention indices, and quantification was performed using the internal standard calibration method. The headspace vial was positioned in the gas chromatograph injection port for automated analysis, and data acquisition was performed using the instrument software [16].

#### 2.3.5. Determination of Monomeric Phenolic Compounds

Wine samples underwent preliminary pretreatment: 2 mL of wine was combined with an equal volume of ethyl acetate, vortexed vigorously, and centrifuged at 5000× *g* for 5 min. The supernatant was carefully transferred to a 15 mL centrifuge tube. This extraction procedure was repeated three times, and the pooled supernatants were evaporated to dryness at 35 °C using a sample rapid evaporation system. The residue was reconstituted in 2 mL of methanol and stored under refrigeration pending chromatographic analysis. Samples were filtered through 0.22 μm microporous membranes immediately prior to HPLC injection.

HPLC analysis was performed on an Agilent 1260 Infinity HPLC system (Agilent Technologies, Santa Clara, CA, USA) equipped with a ShimNex CS C18 column (4.6 × 250 mm, 5 μm, Shimadzu, Kyoto, Japan) at a flow rate of 0.8 mL/min and a column temperature of 30 °C. The mobile phases were (A) water/formic acid (99.9:0.1, *v*/*v*) and (B) acetonitrile/formic acid (99.9:0.1, *v*/*v*). The gradient elution program was: 0–5 min 5% B; 5–35 min 5–35% B; 35–40 min 35–50% B; 40–45 min 50–5% B, followed by a 5 min post-run equilibration. The injection volume was 10 μL, and detection was performed at 280 nm and 320 nm. Quantification was based on external standards (purity ≥ 98%), and the method was adapted from Liu et al. [17] with modifications.

#### 2.3.6. Calculation of Odor Activity Values (OAV)

Odor activity values (OAV) were calculated as the ratio of the mass concentration of each aroma compound to its respective olfactory detection threshold, according to Equation (1):

OAV = C/OT(1)

where C represents the mass concentration of the aroma compound (μg/L), and OT denotes the odor threshold of the respective compound (μg/L). Aroma compounds with OAV ≥ 1 were considered to contribute actively to the overall aromatic profile of the wine.

### 2.4. Statistical Analysis

All experiments were performed with six independent biological replicates, except for aroma analyses, which were conducted in duplicate. Data are expressed as mean ± standard deviation. Statistical analyses were performed using IBM SPSS Statistics 24 software for analysis of variance (ANOVA), followed by Tukey’s HSD post hoc test for multiple comparisons, with statistically significant differences established at *p* < 0.05. Graphical visualizations were generated using the Maiwei Cloud Platform (online version) and R version 4.5.3 (R Core Team, 2024).

## 3. Results

### 3.1. Influence of Fu Tea Addition Level on Basic Physicochemical Indices of Marselan Tea Wine

Fu tea addition progressively decreased alcohol content from 12.24 ± 0.11% (control) to 10.86 ± 0.16% at 1.5 g/L, and modestly reduced residual sugar (Table 1). Volatile acidity remained low (0.44–0.50 g/L) and stable across all treatments, ruling out stuck fermentation. Grape must polyphenols can reduce yeast growth and CO_2_ production and alter nitrogen consumption [18]. Whether such effects occurred in our system—and whether they contributed to the observed decreases—requires further investigation. pH and total acidity were unaffected, ensuring a stable matrix for subsequent analysis.

### 3.2. Influence of Fu Tea Addition on Phenolic Composition and Chromatic Characteristics of Marselan Tea Wine

#### 3.2.1. Changes in Total Phenols, Total Tannins, and Total Anthocyanins

**Figure 1:**
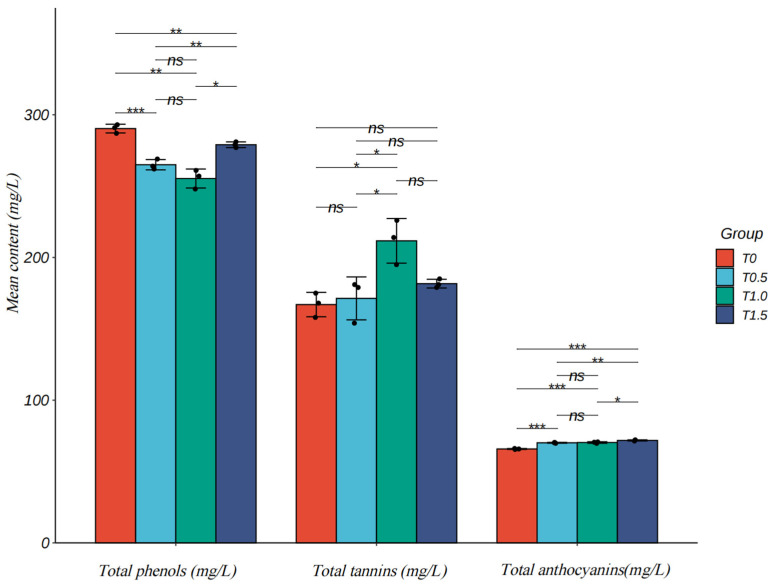
Bar chart illustrating total phenol, total tannin, and total anthocyanin contents in Fu tea–Marselan wine. Data are presented as mean ± SD (n = 6). Significance levels are indicated as follows: * *p* < 0.05, ** *p* < 0.01, *** *p* < 0.001, ns = not significant (*p* > 0.05), based on one-way ANOVA followed by Tukey’s HSD post hoc test.

Total phenols, total tannins, and total anthocyanins all increased significantly (*p* < 0.05) with increasing Fu tea addition (Figure 1). This confirms that Fu tea co-fermentation successfully incorporated tea-derived phenolics into the wine matrix.

The increase in total phenolics was the most direct effect of Fu tea addition. Fu tea is rich in phenolic compounds—including catechins, gallic acid, and their oxidative polymers—which solubilize during fermentation and combine with grape-derived phenolics (proanthocyanidins, flavonols) to form a more complex phenolic matrix.

The increase in total tannins has two implications for wine quality. Tannins reinforce wine structure and astringency, giving the wine more body. They also condense with anthocyanins to form stable polymeric pigments that stabilize color.

The increase in total anthocyanins is central to color improvement. Anthocyanins are the primary chromophores in red wine, but monomeric anthocyanins are unstable and prone to degradation or browning from pH shifts, SO_2_, and oxidation [19]. Fu tea provides abundant polyphenols that can protect and stabilize grape-derived anthocyanins via copigmentation or polymerization, increasing color depth and stability [20]. The observed anthocyanin increase may reflect a combination of direct phenolic input from tea and potential stabilization of grape-derived anthocyanins by tea polyphenols. However, without additional analytical data, we cannot distinguish between these two sources.

#### 3.2.2. Changes in Chromatic Characteristics of Fu Tea–Marselan Wine

All Fu tea-treated groups had significantly higher L* (lightness) values than the control, with L* increasing dose-dependently. This suggests that Fu tea incorporation made the wine brighter and more translucent, possibly due to the clarifying action of tea polyphenols or their interactions with wine pigments (Table 2).

The a* value (red-green coordinate) was positive for all samples, confirming that the wines remained in the red spectrum [21]. However, a* decreased significantly with increasing Fu tea addition compared to the control. This appears to conflict with the increase in total anthocyanins, but actually reflects changes in anthocyanin speciation. Monomeric anthocyanins are vivid red, but polymerization with tannins and other phenolics produces pigments that shift toward orange-red or brick-red hues, lowering a* [22]. These polymeric pigments are typically more stable and less sensitive to pH and SO_2_ [23].

The b* value (yellow-blue coordinate) was significantly lower in all treated groups than in the control, and decreased dose-dependently with Fu tea addition [24]. This further confirms that the wine color shifted toward the red spectrum, with yellow undertones suppressed. Physicochemically, this reflects enhanced blue-light absorption and reduced yellow-light reflectance from progressive anthocyanin polymerization.

Chroma (C) decreased significantly after Fu tea addition, with no significant differences among the addition levels. This reduction may reflect changes in polyphenolic complex formation. When anthocyanins polymerize with tannins and other phenolics, their absorption maxima typically shift bathochromically, reducing color saturation (C). Nevertheless, this polymerization increases pigment stability, which benefits long-term aging and quality consistency.

Hue angle (h°) decreased significantly with increasing Fu tea addition. Together with the declines in a* and b*, this indicates that Fu tea incorporation shifted the wine hue from orange-red (control) toward brick-red or deep red tones—hues associated with aging potential.

ΔE* values exceeded 1.5 for all treated groups and increased with Fu tea addition; all treatment groups differed significantly from the control. ΔE* > 1.5 is perceptible to the human eye. Thus, Fu tea addition caused visually discernible, dose-dependent color changes.

In summary, Fu tea incorporation enhanced L, intensified a, reduced b, shifted h° toward brick-red hues, and while C decreased, pigment stability increased. These color changes are consistent with the increased total phenols, total tannins, and total anthocyanins shown in Figure 1.

#### 3.2.3. Correlation Analysis Between Total Phenols and Hue Angle (h°) in Fu Tea–Marselan Wine

**Figure 2:**
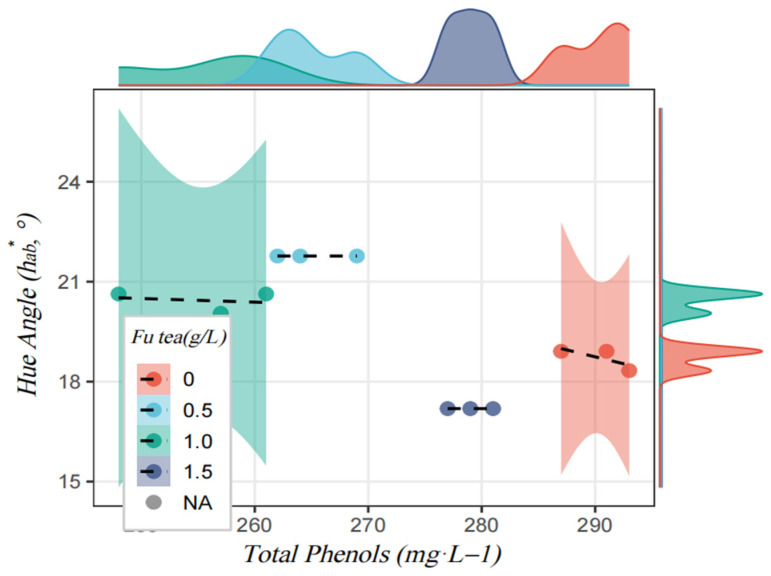
Marginal scatter plot of total phenol content vs. hue angle (h°). Note: The scatter plot shows a linear negative correlation between total phenol content and h° (r = −0.656, *p* = 0.021, n = 12). The black dashed line represents the linear regression fit; the gray shaded area denotes the 95% confidence interval (95% CI: −0.894 to −0.132). Density curves on the top and right axes show the distributions of total phenol content and h° for each treatment group. Color coding: 0 g/L (brick red), 0.5 g/L (lake blue), 1.0 g/L (mint green), 1.5 g/L (navy blue).

To examine the relationship between phenolic content and wine color, we performed Pearson correlation analysis between total phenol content and hue angle (h°) (Figure 2). We found a significant negative correlation between total phenol content and h° (r = −0.656, *p* = 0.0205): higher total phenol content was associated with smaller hue angles, corresponding to a shift toward brick-red/purple-red tones. Linear regression showed that each 100 mg/L increase in total phenolics corresponded to an average decrease of approximately 8° in h°. As Fu tea addition increased from 0 to 1.5 g/L, the distribution of total phenolics shifted rightward (toward higher values), while h° shifted leftward (toward lower values), consistent with Fu tea addition being associated with both phenolic enrichment and hue displacement.

Chlorogenic acid—the most abundant phenolic compound in Fu tea—showed no significant univariate correlation with h° (r = −0.466, *p* = 0.127). This highlights that the shift in hue is not attributable to any single phenolic compound, but is consistent with synergistic effects among multiple tea-derived phenolics. Total phenolic content, as a comprehensive measure of the phenolic matrix, thus predicted chromatic changes better than any individual monomeric phenol.

#### 3.2.4. Principal Component Analysis (PCA) of Phenolic and Color Parameters

**Figure 3:**
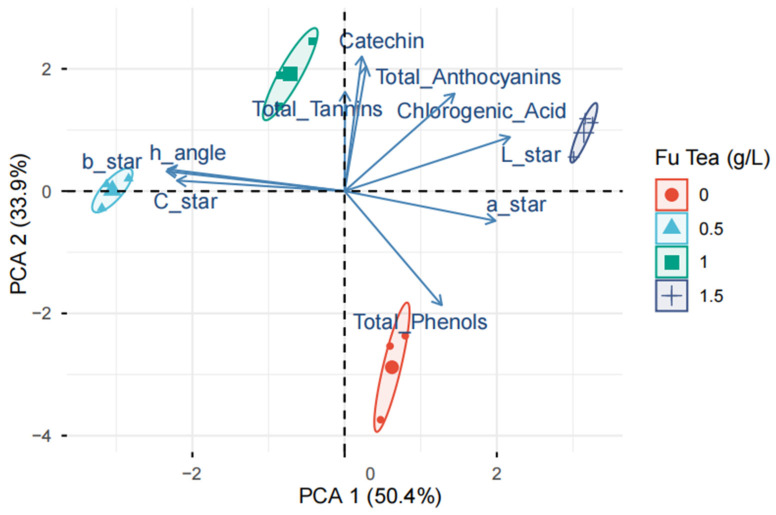
PCA biplot of phenolic compounds and color parameters. Samples are colored by treatment group, with 95% confidence ellipses. Arrows represent variable loadings; their direction and length indicate the contribution to PC1 and PC2.

We performed principal component analysis (PCA) to investigate the multivariate relationships between phenolic composition and color parameters (Figure 3). PC1 and PC2 accounted for 50.45% and 33.89% of the total variance, respectively (cumulative 84.34%). Variable contribution analysis (Table 3) showed that PC1 was dominated by color parameters: b* (19.38%), h° (19.14%), Cab (17.19%), and L (16.73%). PC2 mainly captured variation in the phenolic profile, with catechin (25.59%), total anthocyanins (21.96%), total phenols (18.36%), and total tannins (13.90%) as the key contributors.

Chlorogenic acid was the only phenolic compound with notable contributions to both PC1 (7.39%) and PC2 (13.44%). This dual contribution suggests an association between chlorogenic acid and both the color-related (PC1) and phenolic-related (PC2) variation. The PCA score plot showed clear separation of samples along PC1 by Fu tea dosage: the control group clustered on the negative side and the 1.5 g/L group on the positive side, consistent with a dose-dependent shift in color characteristics.

### 3.3. Monomeric Phenolic Composition of Fu Tea–Marselan Wine

We quantified 18 monomeric phenolic compounds by HPLC across different Fu tea addition levels. To investigate the effect of Fu tea addition on the phenolic composition, we applied orthogonal partial least squares discriminant analysis (OPLS-DA), variable importance in projection (VIP) analysis, and Mfuzz clustering to identify key differential metabolites and characterize their variation patterns.

#### 3.3.1. OPLS-DA Analysis of Monomeric Phenolic Composition Differences

As shown in Table 4, the monomeric phenols detected in Marselan wine comprised mainly phenolic acids, flavanols, and flavonols. Chlorogenic acid, 3,4-dihydroxybenzoic acid (protocatechuic acid), and salicylic acid were present at relatively high concentrations in all sample types.

**Figure 4:**
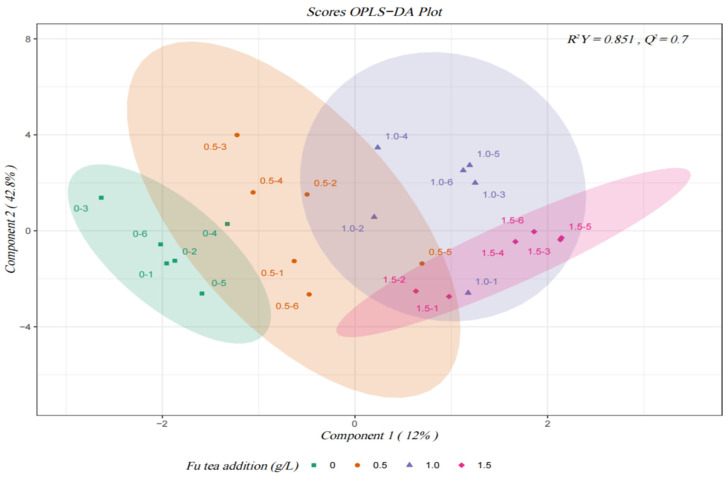
OPLS-DA score plot of monomeric phenolic compounds across different Fu tea addition levels. Note: Model parameters: R^2^Y = 0.851, Q^2^ = 0.7. The predictive component (Component 1, 12%) accounts for the separation trend among samples with different tea addition levels, while the orthogonal component (Component 2, 42%) reflects within-group variability. Each point represents a biological replicate (n = 6), and ellipses denote 95% confidence intervals. Permutation testing (200 iterations) indicated no model overfitting (Supplementary Figure S1).

To further investigate the effect of Fu tea addition on the phenolic composition, we quantified 18 monomeric phenols by HPLC and constructed an OPLS-DA model (Figure 4). The model parameters (R^2^Y = 0.851, Q^2^ = 0.7) indicate good explanatory and predictive performance. Permutation testing (200 iterations) indicated no overfitting (Supplementary Figure S1).

The OPLS-DA score plot (Figure 4) shows clear, statistically significant separation of samples from different treatment groups. Control samples (0 g/L) clustered on the left side of the plot; Fu tea-treated groups were located on the right side. Sample points shifted progressively rightward along Component 1 with increasing Fu tea addition. The 95% confidence ellipses of the 1.0 g/L and 1.5 g/L groups were fully separated from the control, indicating that higher Fu tea dosages induced systematic changes in the monomeric phenolic composition. These changes were evident in both the types and relative abundances of phenolic compounds.

#### 3.3.2. Identification and Variation Patterns of Key Differential Monomeric Phenols

**Figure 5:**
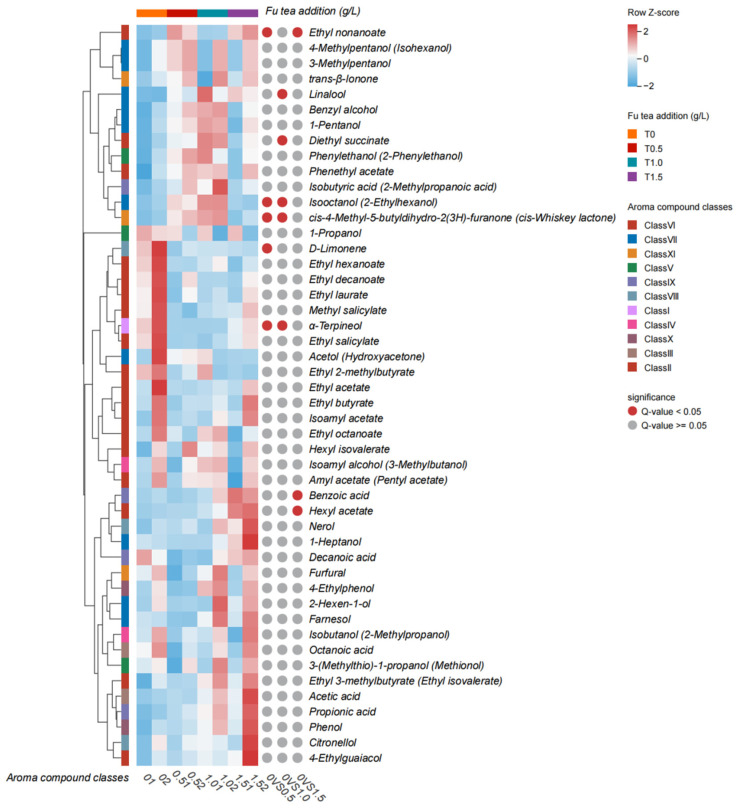
Heatmap of volatile compounds in Fu tea–Marselan wine.

To identify the key phenolic compounds contributing to intergroup differentiation, we calculated VIP values for each metabolite and visualized them in a heatmap (Figure 5). Metabolites with VIP > 1 were designated as key differential compounds. Chlorogenic acid (highest VIP), catechin, myricetin, protocatechuic acid, and salicylic acid were identified as the compounds with the highest contribution to group differentiation.

#### 3.3.3. Mfuzz Clustering of Differential Phenolic Compounds

**Figure 6:**
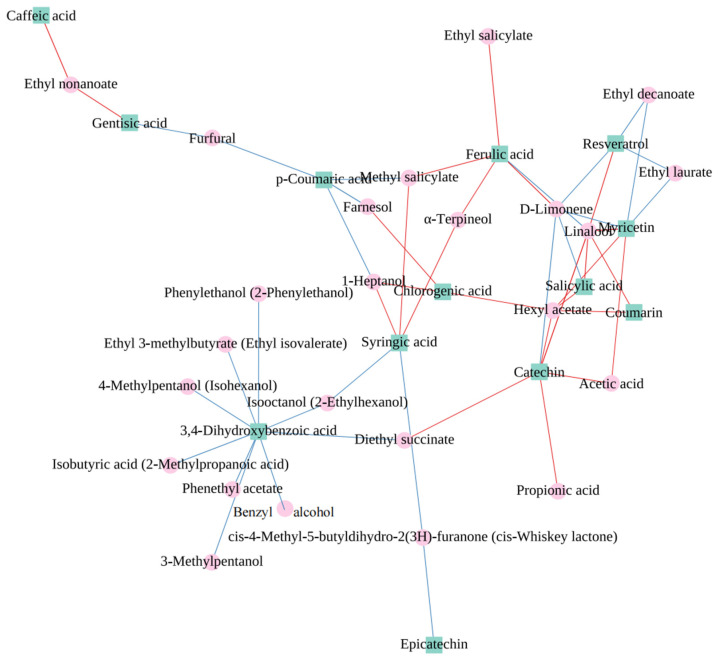
Correlation network of phenolic compounds and key aroma-active substances (OAV ≥ 1). Green nodes represent phenolic compounds; pink nodes represent aroma compounds. Edge thickness is proportional to the absolute value of the Pearson correlation coefficient (|r| > 0.6, *p* < 0.05). Red edges indicate positive correlations; blue edges indicate negative correlations. Node size reflects the degree of connectivity. Chlorogenic acid and catechin showed the highest connectivity, linking to both positively and negatively correlated aroma compounds.

To further characterize the dynamic variation patterns of key phenolic compounds in response to Fu tea addition, we performed Mfuzz clustering analysis (Figure 6). The differentially abundant phenolic compounds (VIP > 1) were categorized into six distinct variation patterns. Subclasses 4 and 6 showed the most representative trends. Subclass 4 contained chlorogenic acid, catechin, and myricetin, whose normalized concentrations increased sustainedly with Fu tea addition. Chlorogenic acid—a hydroxycinnamic acid derivative—is a known antioxidant with free radical scavenging and anti-inflammatory activities [25]. Catechin, the most abundant flavan-3-ol in tea, contributes to wine color stability and astringency. In the wine matrix, it can engage in copigmentation or condensation reactions with anthocyanins, forming more stable polymeric pigments [26]. Myricetin, a flavonol, also exhibits antioxidant activity. These three metabolites are characteristic or abundant phenolic constituents of Fu tea. Their coordinated increase shows the successful leaching and integration of tea-derived phenolics into the wine matrix, with incorporation positively correlated with Fu tea addition level.

In contrast, 3,4-dihydroxybenzoic acid (protocatechuic acid) in Subclass 1 showed a declining trend with Fu tea addition. This may reflect consumption of protocatechuic acid in polymerization or oxidation reactions involving other phenolic compounds introduced by Fu tea. Protocatechuic acid has a molecular structure conducive to oxidative polymerization, especially in the presence of other phenolics and polyphenol oxidase [27]. This decline, together with the observed color shift toward brick-red hues, suggests that protocatechuic acid may participate in complexation or condensation reactions that generate more stable polymeric pigments.

### 3.4. Aroma Composition Analysis of Fu Tea–Marselan Wine

#### 3.4.1. Overall Changes in Volatile Compound Composition

We detected and identified 78 volatile compounds by HS-SPME-GC-MS, including alcohols, esters, aldehydes/ketones, acids, and terpenes. A clustering heatmap (Figure 5) was constructed to visualize the influence of Fu tea addition on volatile composition. The control group (0 g/L) and all Fu tea-treated groups showed markedly distinct volatile profiles, forming two independent clusters. This indicates that Fu tea incorporation restructured the overall aroma profile of Marselan wine. Among the tea-treated groups, the 1.0 g/L and 1.5 g/L treatments clustered together first, then merged with the 0.5 g/L group, a pattern suggesting dose-dependent modulation of aroma composition (Table 5).

Based on chemical classification, the clustering dendrogram on the right separated the volatile compounds into two main clusters. Cluster I predominantly contained esters (e.g., ethyl acetate, ethyl hexanoate) and higher alcohols (e.g., isoamyl alcohol, 2-phenylethanol), key contributors to fruity and floral aromas. In Fu tea-treated groups, the relative abundances of these compounds were generally higher than in the control (darker coloration in the heatmap). Enhanced ester accumulation is often associated with increased secondary metabolism of *Saccharomyces cerevisiae*, especially in phenolic-containing fermentation environments [29]. Cluster II mainly comprised aldehydes and C6 alcohols (e.g., 1-hexanol, cis-3-hexen-1-ol), which were more abundant in the control and declined with increasing Fu tea addition. C6 alcohols are typically linked to herbaceous and vegetal notes. Their reduction may be related to the antioxidant activity of Fu tea phenolics, which could inhibit lipoxygenase and reduce formation of these precursors [26].

In the 1.0 g/L and 1.5 g/L groups, several characteristic Fu tea aroma compounds were detected at elevated levels or as newly identified compounds. For example, 1-octen-3-ol (mushroom-like, earthy)—a key metabolite of *E. cristatum*—is recognized as a core contributor to the “fungal floral” aroma of Fu tea [30]. Linalool, an important terpene, also increased significantly and contributes floral and citrus-like notes. Its increase may reflect leaching of terpene precursors from Fu tea, or modulation of terpene biosynthetic pathways in *S. cerevisiae* [31]. Certain lactones (coconut-like, creamy) also emerged, further enriching the aroma spectrum. The presence of these characteristic compounds is consistent with the involvement of Fu tea microbial metabolites in the co-fermentation process, contributing to the aromatic complexity of the wine.

In summary, the heatmap analysis shows that Fu tea addition remodeled the volatile composition of Marselan wine. Higher addition levels introduced tea-characteristic aroma constituents while enhancing fruity and floral compounds, resulting in a marked increase in aromatic complexity.

#### 3.4.2. Differential Analysis of Key Odor-Active Compounds

To quantitatively assess the actual contribution of individual volatile compounds to the overall aromatic profile of Marselan tea wine, odor activity values (OAV) were calculated. Based on aroma-active compounds with OAV > 1, volcano plot analysis was performed (Figure S4) to identify key differential aroma compounds among treatment groups.

Analysis revealed that, relative to the control (0 g/L Fu tea), a total of 23 aroma-active compounds (OAV > 1) underwent significant alterations in the Fu tea-supplemented groups, with 19 compounds exhibiting pronounced upregulation and four compounds displaying significant downregulation. Prominently upregulated aroma compounds included: 1-octen-3-ol, imparting a characteristic mushroom-like aroma; linalool and 2-phenylethanol, contributing floral notes; ethyl isobutyrate and ethyl hexanoate, conferring fruity characteristics reminiscent of strawberry and pineapple; and ethyl 2-hydroxypropanoate, contributing creamy nuances. The synergistic enhancement of these key aroma compounds facilitated a transition in the aromatic profile of Marselan tea wine from a relatively simplistic fruity foundation toward a more complex, multi-layered composite aroma integrating floral, fruity, distinctive fungal, and creamy notes. Among these, the marked elevation in OAV of 1-octen-3-ol, a signature metabolite of *E. cristatum* in Fu tea, provides direct and compelling evidence for the unique contribution of Fu tea to the flavor profile of the co-fermented wine [32]. The increased abundance of ester compounds reflects enhanced esterification of higher alcohols and fatty acids by *S. cerevisiae* within the co-fermentation milieu, potentially attributable to certain Fu tea constituents acting as aroma precursors or metabolic modulators influencing yeast ester synthesis pathways [33].

Conversely, significantly downregulated aroma compounds primarily included 2-hexenal (grassy, green leaf aroma) and 1-heptanol (herbaceous aroma). These C6 compounds are generally associated with incomplete grape ripening or oxidative reactions occurring during crushing, and their diminished levels suggest that the co-fermentation process may effectively optimize wine aroma quality by substantially reducing undesirable herbaceous notes. This outcome aligns with the potent antioxidant activity exhibited by the abundant phenolic compounds in Fu tea, which may suppress C6 compound biosynthesis through inhibition of lipoxygenase activity [34]. Notably, comparison of the volcano plots for 1.5 g/L vs. 0 g/L and 1.0 g/L vs. 0 g/L revealed that at excessively high Fu tea addition (1.5 g/L), the upregulation magnitude of certain aroma compounds was attenuated, and in some instances, suppression was observed. This phenomenon may be related to the inhibitory effects of high phenolic concentrations on the metabolic activity or key enzymatic functions of *S. cerevisiae;* for instance, phenolic compounds may bind to yeast cell wall components [33] or directly inhibit the activity of enzymes involved in aroma compound biosynthesis, thereby modulating the biogenesis of volatile metabolites.

#### 3.4.3. Variation Pattern Analysis of Key Odor-Active Compounds

To further characterize the variation patterns of different aroma compounds in response to Fu tea addition, we applied K-means clustering analysis to aroma-active compounds with OAV > 1 (Figure S5). The clustering results categorized these compounds into several distinct variation trajectories. Subclasses 4, 6, and 9 represented compounds with sustained increasing trends, including 1-octen-3-ol, linalool, and various ethyl esters. The OAV values of these compounds increased continuously with Fu tea addition, suggesting that they are major contributors to the complex aroma profile of Marselan tea wine.

The increases in these compounds could be partly explained by the release of aroma precursors from Fu tea, or by the action of β-glucosidase—an enzyme known to be produced by *E. cristatum* in tea fermentation [35]—which may hydrolyze glycosidically bound aroma precursors. However, these possibilities require experimental verification in our system. Other subclasses showed sustained declining trends, exemplified by 2-hexenal, whose OAV decreased progressively with increasing Fu tea addition. This reduction may be associated with the antioxidant activity of tea-derived phenolics, which could inhibit fatty acid oxidation pathways and reduce the formation of C6 compounds [36]. This could potentially contribute to a reduction in herbaceous notes, though sensory evaluation would be required to confirm this effect.

In summary, K-means clustering analysis revealed distinct variation patterns of aroma-active compounds in response to Fu tea addition. The data suggest that Fu tea addition was associated with both increased levels of positive aroma compounds and decreased levels of herbaceous off-flavors.

### 3.5. Correlation Analysis Between Phenolic Compounds and Volatile Aroma Constituents

To further investigate the associations between phenolic compound alterations and changes in the volatile profile, we performed Pearson correlation analysis between monomeric phenol contents and aroma-active compounds (OAV > 1). The results are visualized in a correlation heatmap (Figure S6) and a correlation network (Figure 6).

#### 3.5.1. Correlation Heatmap Analysis

We performed Pearson correlation analysis between volatile compounds (OAV > 1) and monomeric phenols. The results are presented as a correlation heatmap (Figure S6), which shows extensive and complex correlations between phenolic compounds and aroma constituents. Specifically, chlorogenic acid, catechin, and myricetin—three phenolic compounds whose contents increased significantly with increasing Fu tea addition—showed highly significant positive correlations with several key aroma compounds, including 1-octen-3-ol, linalool, and hexyl acetate. This finding indicates that the integration of tea-derived phenolic compounds is associated with the formation of Fu tea-characteristic aromas (e.g., 1-octen-3-ol) as well as fruity and floral esters.

Such positive correlations may have multiple explanations. First, Fu tea inherently provides abundant aroma precursors and phenolic compounds, which are released during co-fermentation. Second, certain phenolic compounds may influence microbial metabolism and thereby promote the biosynthesis of specific aroma compounds [37]. However, our correlative data do not allow us to distinguish among these possibilities.

Conversely, herbaceous aroma compounds that decreased with Fu tea addition—such as 2-hexenal and 1-heptanol—showed significant negative correlations with tea-derived phenolics, including chlorogenic acid and catechin. This observation is consistent with the hypothesis that tea-derived phenolics may contribute to the reduction in herbaceous off-flavors, potentially through their antioxidant activity [38,39]. However, direct evidence (e.g., LOX activity assays, inhibitor controls) is needed to confirm this mechanism.

#### 3.5.2. Correlation Network Analysis of Phenolic and Aroma Compounds

Based on significant correlations (|r| > 0.6, *p* < 0.05), we constructed a correlation network (Figure 6). Green nodes represent monomeric phenolic compounds, and pink nodes represent aroma-active compounds. The thickness of the connecting edges is proportional to the absolute value of the correlation coefficient, with red edges indicating positive correlations and blue edges indicating negative correlations. The size of each node reflects its degree of connectivity within the network.

Chlorogenic acid and catechin showed the highest degree of connectivity in the network, linking to a wide array of aroma compounds. The network showed a natural separation into two main clusters based on correlation sign and strength. Cluster 1 comprised tea-derived phenolics (e.g., chlorogenic acid, catechin) and desirable aroma compounds, including 1-octen-3-ol (mushroom-like), linalool (floral), and various ethyl esters (fruity). These connections were predominantly characterized by thick red edges, reflecting strong positive correlations. Cluster 2 consisted of phenolic compounds and herbaceous off-flavor compounds, such as 2-hexenal and 1-heptanol. These associations were depicted by thick blue edges, indicating significant negative correlations.

These two clusters are interconnected through the core phenolic nodes, forming a complex regulatory network. The correlation network is consistent with a model in which tea-derived phenolics are associated with both the accumulation of desirable aroma compounds and the reduction in herbaceous off-flavors. However, causality cannot be inferred from this correlative network analysis.

## 4. Discussion

This study provides the first comprehensive mechanistic investigation of Marselan wine co-fermented with Fu brick tea. Our findings show that the quality enhancement does not come from a single “star molecule,” but from a multifaceted phenolic network synergy orchestrated by tea-derived compounds.

The decreased alcohol and residual sugar—together with stable volatile acidity—are consistent with a potential modification of carbon flux in *S. cerevisiae.* However, we lack direct evidence (e.g., yeast biomass, sugar consumption kinetics). Mekoue Nguela et al. [13] reported that grape must polyphenols reduce yeast growth and CO_2_ production and alter nitrogen consumption. Although these parameters were not directly measured in our study, this mild metabolic stress has been suggested to divert glycolytic intermediates toward esters and higher alcohols, enriching wine flavor without compromising fermentation stability [21]. Notably, fermentation duration (12–14 days) was similar across all treatments, suggesting that tea addition did not substantially prolong or inhibit the fermentation process.

The presence of diverse phenolic classes—including hydroxycinnamic acids (chlorogenic acid, caffeic acid), flavan-3-ols (catechin), and flavonols (myricetin, quercetin)—suggests a synergistic effect on color stabilization. The co-fermentation with Fu tea introduced additional phenolic compounds that likely participated in copigmentation and polymerization reactions with grape-derived anthocyanins. The concentration-dependent effects observed in this study indicate that the phenolic network is finely balanced: at 1.0 g/L, the enhancement was optimal, while at 1.5 g/L, some aroma compounds showed attenuated upregulation, suggesting a possible threshold effect for phenolic-mediated aroma modulation.

The improvement in color—especially the decreased h° and b* and the increased total anthocyanins—is a classic indicator of enhanced copigmentation and polymerization. Tea-derived phenolic acids, especially chlorogenic acid, act as efficient cofactors that engage in π–π stacking with the flavylium cation of monomeric anthocyanins [11,16]. This non-covalent interaction protects the chromophore from water attack, causing a bathochromic shift and intensifying color, manifesting as lower a, b, and h° [17,18]. Tea-derived catechins can also covalently polymerize with anthocyanins, forming more stable polymeric pigments that enhance long-term color stability [5].

A key finding is the significant negative correlation between total phenolic content and h° (r = –0.656, *p* = 0.0205), while chlorogenic acid alone showed no significant correlation. This underscores that copigmentation efficiency depends on molar ratios and structural compatibility of multiple cofactors, not on the absolute concentration of any single compound [18]. Total phenolic content thus serves as a more robust proxy for the combined effects of the entire phenolic network. PCA further clarified chlorogenic acid’s unique role. It was the only compound contributing significantly to both PC1 (color) and PC2 (phenolics). This dual role arises from chlorogenic acid’s structure. The caffeoyl moiety facilitates π–π stacking with anthocyanins (PC1). The quinic acid backbone enhances solubility and integration into the phenolic matrix (PC2) [40].

Fu tea co-fermentation profoundly reshaped the volatile aroma profile. The detection of 1-octen-3-ol—a signature metabolite of *E. cristatum* [6,19]—directly demonstrates that the tea’s fungal flora contributes to wine aroma complexity. The increase in esters and higher alcohols—including phenylethyl alcohol and isoamyl acetate—enhances fruity and floral notes. This may reflect tea polyphenols’ influence on yeast acetyltransferase activity, which promotes ester biosynthesis [21]. Conversely, the reduction in C6 compounds—which give herbaceous or “green” notes—may result from tea polyphenols’ antioxidant activity. They may inhibit lipoxygenase (LOX), which oxidizes unsaturated fatty acids [20]. This dual effect—enhancing desirable aromas while suppressing undesirable ones—represents a powerful “gain and loss” optimization of the wine’s aromatic landscape.

Compared to grape stems—which primarily add tannins and can increase astringency [9]—Fu tea offers a more balanced mix of phenolic acids, flavanols, and unique microbial metabolites. This makes Fu tea a particularly attractive candidate for producing differentiated wines with enhanced aging potential and a complex, multi-layered flavor profile.

### Limitations

This study provides robust chemical and statistical evidence for these mechanisms, but is limited by the lack of direct microbial community analysis and quantitative sensory evaluation. We also did not perform model–solution copigmentation assays or yeast enzyme activity measurements. The results derive from a single harvest and region; extrapolation to other vintages, regions, or scales requires further validation. Future work should use metatranscriptomics to elucidate cross-kingdom interactions between *S. cerevisiae* and *E. cristatum*, and integrate trained sensory panels to link instrumental data with human perception.

## 5. Conclusions

Co-fermenting Marselan grapes with Fu brick tea is a highly effective and innovative strategy for enhancing wine quality. Under the specific conditions of this study (Yanqi Basin, 2023 harvest, laboratory scale), adding 1.0 g/L Fu tea gave the most favorable improvement in color stability and aroma complexity; addition above this level was associated with diminishing or negative effects on certain aroma compounds. The key mechanism is a multifaceted phenolic network centered on chlorogenic acid, which is associated with color enhancement through copigmentation and polymerization, and reshapes the aroma profile by promoting desirable esters and suppressing herbaceous off-flavors. Specifically, the major phenolic compounds identified in the tea-wine included chlorogenic acid, catechin, and myricetin, while the key volatile compounds contributing to aroma complexity included 1-octen-3-ol, linalool, and various ethyl esters. However, causality remains to be established through targeted experiments (e.g., model–solution copigmentation assays, yeast enzyme activity measurements, microbial community analysis). This study provides a preliminary theoretical and practical reference for developing tea-wine products, and offers new avenues for high-value use of Fu tea resources.

## Figures and Tables

**Table 1:** Basic physicochemical indices of Fu tea–Marselan wine.

Fu Tea Addition Level (g/L)	Alcohol Content (%Vol)	Volatile Acidity (g/L)	Residual Sugar (g/L)	pH Value	Total Acidity (g/L)	Glycerol (g/L)
0 (Control)	12.24 ± 0.11 ^a^	0.50 ± 0.00 ^a^	1.36 ± 0.04 ^a^	3.89 ± 0.06 ^a^	5.73 ± 0.03 ^a^	9.37 ± 0.77 ^a^
0.5	11.81 ± 0.39 ^a^	0.47 ± 0.33 ^a^	1.29 ± 0.03 ^ab^	3.91 ± 0.23 ^a^	5.67 ± 0.18 ^a^	9.41 ± 0.32 ^a^
1.0	11.71 ± 0.23 ^ab^	0.47 ± 0.33 ^a^	1.21 ± 0.01 ^b^	3.90 ± 0.14 ^a^	5.53 ± 0.09 ^a^	9.24 ± 0.21 ^a^
1.5	10.86 ± 0.16 ^b^	0.44 ± 0.31 ^a^	1.23 ± 0.03 ^b^	3.88 ± 0.07 ^a^	5.40 ± 0.10^a^	8.87 ± 0.17 ^a^

Note: ANOVA revealed a significant effect of Fu tea addition on alcohol content (F(3, 8) = 7.67, *p* < 0.01) and residual sugar (F(3, 8) = 10.02, *p* < 0.001), while volatile acidity, pH, total acidity, and glycerol were not significantly affected (*p* > 0.05). Different superscript letters in the same column indicate significant differences (*p* < 0.05).

**Table 2:** Chromatic parameters of Fu tea–Marselan wine.

Parameter	Fu Tea Addition Level (g/L)			
	0 (Control)	0.5	1.0	1.5
L*	14.62 ± 0.24 ^a^	16.16 ± 0.04 ^b^	16.28 ± 0.01 ^b^	16.42 ± 0.01 ^b^
a*	3.48 ± 0.08 ^a^	2.77 ± 0.01 ^b^	2.77 ± 0.06 ^b^	2.64 ± 0.02 ^b^
b*	1.35 ± 0.09 ^a^	0.76 ± 0.03 ^b^	0.52 ± 0.02 ^c^	0.35 ± 0.03 ^c^
$C_{ab}^{*}$	3.74 ± 0.10 ^a^	2.87 ± 0.01 ^b^	2.82 ± 0.04 ^b^	2.66 ± 0.02 ^b^
$h_{ab}^{*}$	21.09 ± 1.08 ^a^	15.29 ± 0.54 ^b^	10.61 ± 0.29 ^c^	7.56 ± 0.51 ^d^
*∆* $E_{ab}^{*}$	0.00 ± 0.02 ^a^	1.80 ± 0.23 ^b^	1.99 ± 0.04 ^b^	2.22 ± 0.05 ^b^

Note: One-way ANOVA revealed that Fu tea addition significantly affected all chromatic parameters: L* (F(3, 20) = 229, *p* < 0.001), a* (F(3, 20) = 272, *p* < 0.001), b* (F(3, 20) = 397, *p* < 0.001), C* (F(3, 20) = 402, *p* < 0.001), h° (F(3, 20) = 436, *p* < 0.001), and ΔE* (F(3, 20) = 423, *p* < 0.001). Different superscript letters in the same column indicate significant differences (*p* < 0.05).

**Table 3:** Variable contributions to the first two principal components (PC1 and PC2).

Variable	PC1 Contrib (%)	PC2 Contrib (%)
b*	19.38	0.55
$h_{ab}^{*}$	19.14	0.67
$C_{ab}^{*}$	17.19	0.16
L*	16.73	4.12
a*	13.92	1.24
Chlorogenic Acid	7.39	13.44
Total Phenols	5.78	18.36
Total Anthocyanins	0.29	21.96
Catechin	0.18	25.59
Total Tannins	0	13.9

**Table 4:** Monomeric phenolic composition (mg/L) of Fu tea–Marselan wine.

Monomeric Phenols (mg/L)	Fu Tea Addition Level (g/L)			
	0 (Control)	0.5	1.0	1.5
Gallic acid	0.0125 ± 0.003 ^a^	0.0111 ± 0.001 ^a^	0.0089 ± 0.001 ^c^	0.0111 ± 0.001 ^a^
3,4-Dihydroxybenzoic acid	0.4275 ± 0.174 ^a^	0.252 ± 0.040 ^b^	0.2312 ± 0.037 ^b^	0.2577 ± 0.023 ^b^
Gentisic acid	0.0432 ± 0.012 ^a^	0.0439 ± 0.007 ^a^	0.0331 ± 0.005 ^a^	0.0422 ± 0.004 ^a^
Chlorogenic acid	0.7591 ± 0.256 ^a^	0.8341 ± 0.503 ^a^	1.2122 ± 0.149 ^b^	1.4154 ± 0.108 ^c^
Vanillic acid	0.1337 ± 0.055 ^a^	0.1183 ± 0.018 ^a^	0.1053 ± 0.014 ^b^	0.1299 ± 0.011 ^a^
Caffeic acid	0.1552 ± 0.048 ^a^	0.1688 ± 0.026 ^b^	0.1338 ± 0.021 ^b^	0.1645 ± 0.014 ^b^
Syringic acid	0.0184 ± 0.002 ^a^	0.0148 ± 0.002 ^b^	0.0137 ± 0.002 ^b^	0.0171 ± 0.001 ^a^
Epicatechin	0.0046 ± 0.001 ^a^	0.0043 ± 0.001 ^a^	0.0034 ± 0.001 ^a^	0.0043 ± 0.001 ^a^
Catechin	0.0580 ± 0.014 ^a^	0.1016 ± 0.031 ^b^	0.0872 ± 0.014 ^c^	0.0916 ± 0.012 ^c^
p-Coumaric acid	0.1384 ± 0.020 ^a^	0.2546 ± 0.122 ^b^	0.1132 ± 0.007 ^a^	0.1353 ± 0.010 ^a^
Ferulic acid	0.1486 ± 0.080 ^a^	0.1093 ± 0.022 ^b^	0.0945 ± 0.024 ^c^	0.1008 ± 0.011 ^c^
Salicylic acid	0.3273 ± 0.021 ^a^	0.4009 ± 0.083 ^b^	0.3445 ± 0.039 ^a^	0.3364 ± 0.073 ^a^
Rutin	0.0155 ± 0.002 ^a^	0.0144 ± 0.002 ^a^	0.0383 ± 0.040 ^b^	0.0151 ± 0.002 ^a^
Coumarin	0.0336 ± 0.002 ^a^	0.0355 ± 0.006 ^a^	0.0329 ± 0.006 ^a^	0.0360 ± 0.005 ^a^
Phloridzin	0.0387 ± 0.004 ^a^	0.0249 ± 0.015 ^a^	0.0177 ± 0.008 ^b^	0.0198 ± 0.006 ^b^
Myricetin	0.0214 ± 0.002 ^a^	0.0253 ± 0.004 ^a^	0.0237 ± 0.004 ^a^	0.0251 ± 0.003 ^a^
Resveratrol	0.0619 ± 0.005 ^a^	0.0720 ± 0.012 ^a^	0.0653 ± 0.012 ^a^	0.0704 ± 0.009 ^a^
Quercetin	0.0731 ± 0.009 ^a^	0.4402 ± 0.010 ^b^	0.0700 ± 0.009 ^a^	0.0736 ± 0.007 ^a^

Note: One-way ANOVA revealed significant effects of Fu tea addition on chlorogenic acid (F(3, 20) = 6.609, *p* = 0.003), catechin (F(3, 20) = 5.764, *p* = 0.005), and p-coumaric acid (F(3, 20) = 6.346, *p* = 0.003). Different superscript letters in the same column indicate significant differences (*p* < 0.05).

**Table 5:** Aroma composition of Fu tea–Marselan wine (μg/L).

Aroma Compounds	OT [26,28] (μg/L)	Fu Tea Addition Level (g/L)			
		0 (Control)	0.5	1.0	1.5
**Varietal Aroma Compounds**					
**Terpene Alcohol**					
α-Terpineol	6.00	39.25 ± 14.43	6.26 ± 0.06	6.33 ± 0.22	22.73 ± 5.94
**Volatile Phenol**					
4-Ethylguaiacol	22.00	151.11 ± 0.01	151.11 ± 0.01	151.1183 ± 0.01	151.1387 ± 0.02
**Fatty Acids**					
Octanoic acid	500.00	763.06 ± 19.01	724.83 ± 21.32	743.72 ± 18.58	751.37 ± 38.52
Acetic acid	200,000.00	112,212.50 ± 21,990.42	145,420.99 ± 10,852.38	290,764.09 ± 79,099.14	375,879.18 ± 198,191.23
**Higher Alcohols**					
Isoamyl alcohol (3-Methylbutanol)	30,000.00	244,586.81 ± 82,619.29	188,074.99 ± 91,541.84	300,821.11 ± 7078.53	194,249.77 ± 119,325.18
Isobutanol (2-Methylpropanol)	40,000.00	65,448.13 ± 17,955.34	48,483.53 ± 10,862.36	60,527.49 ± 13,184.02	58,745.68 ± 40,279.53
**Alcohols**					
Phenylethanol (2-Phenylethanol)	14,000.00	19,247.83 ± 6885.76	35,786.21 ± 5279.80	35,800.37 ± 9146.39	24,841.41 ± 8495.65
3-(Methylthio)-1-propanol (Methionol)	1000.00	2175.89 ± 132.47	1909.18 ± 594.35	2269.56 ± 614.59	2223.5646 ± 445.8598
1-Propanol	50,000.00	73,384.56 ± 4836.65	60,291.02 ± 12,678.92	56,093.41 ± 22,210.00	59,596.51 ± 20,179.33
**Esters**					
Isoamyl acetate	30.00	342.73 ± 146.14	270.71 ± 11.54	300.89 ± 60.75	358.01 ± 101.47
Ethyl hexanoate	14.00	472.60 ± 183.16	106.11 ± 7.58	225.33 ± 75.09	99.01 ± 114.46
Ethyl butyrate	20.00	175.37 ± 58.90	127.17 ± 13.73	142.82 ± 9.93	167.7160 ± 64.7130
Ethyl acetate	7500.00	72,600.46 ± 38,670.62	41,452.38 ± 1117.39	46,221.4513 ± 3597.0346	56,796.53 ± 19,083.80
Ethyl 2-methylbutyrate	18.00	225.56 ± 44.46	89.94 ± 32.84	132.13 ± 116.35	57.65 ± 8.83
Ethyl octanoate	874.00	2966.90 ± 1046.28	2269.57 ± 347.16	3216.55 ± 193.32	2073.35 ± 726.75
Hexyl isovalerate	3.00	4.85 ± 1.11	5.44 ± 1.26	5.43 ± 0.25	5.04 ± 1.18
Ethyl 3-methylbutyrate (Ethyl isovalerate)	3.00	4.38 ± 3.31	5.06 ± 0.13	9.92 ± 2.12	9.42 ± 3.66
Ethyl decanoate	200.00	294.97 ± 88.04	178.85 ± 86.60	146.99 ± 1.56	178.68 ± 62.64
**Fermentation-Derived Compounds**					
**Esters**					
Amyl acetate (Pentyl acetate)	30.00	6.28 ± 1.71	5.67 ± 1.05	6.54 ± 0.10	5.21 ± 2.39
Diethyl succinate	6000.00	522.40 ± 194.49	978.71 ± 17.56	1446.94 ± 47.24	840.72 ± 287.43
Ethyl laurate	1500.00	62.61 ± 20.63	35.68 ± 15.57	34.57 ± 4.12	39.94 ± 15.95
Ethyl nonanoate	1300.00	8.70 ± 0.82	18.38 ± 2.33	10.03 ± 0.16	18.73 ± 2.12
Hexyl acetate	670.00	0.08 ± 0.01	0.10 ± 0.00	0.19 ± 0.05	0.46 ± 0.03
Ethyl salicylate	/	4.56 ± 0.15	4.27 ± 0.01	4.28 ± 0.04	4.41 ± 0.05
Methyl salicylate	/	2.30 ± 1.01	0.51 ± 0.26	0.97 ± 0.12	1.57 ± 0.62
Phenethyl acetate	73.00	14.05 ± 3.19	19.20 ± 1.47	19.92 ± 0.29	17.28 ± 4.30
**Alcohols**					
Linalool	200.00	10.06 ± 3.30	116.84 ± 25.59	195.54 ± 96.09	163.83 ± 22.56
1-Pentanol	64,000.00	3168.22 ± 796.52	4734.56 ± 217.80	5530.56 ± 121.18	3821.67 ± 1443.38
Isooctanol (2-Ethylhexanol)	8000.00	14.86 ± 9.69	71.99 ± 6.10	107.67 ± 0.44	19.14 ± 4.99
Benzyl alcohol	200,000.00	866.87 ± 66.23	1015.58 ± 51.25	1083.30 ± 10.99	929.99 ± 85.24
3-Methylpentanol	15,000.00	15.33 ± 16.13	37.35 ± 5.03	23.16 ± 23.10	22.78 ± 17.95
4-Methylpentanol (Isohexanol)	500,000.00	16.06 ± 16.90	39.13 ± 5.27	24.26 ± 24.20	23.87 ± 18.81
2-Hexen-1-ol	/	10,724.80 ± 12,839.96	2101.88 ± 586.05	19,804.01 ± 26,911.29	20,048.74 ± 12,504.35
Farnesol	/	1389.71 ± 151.71	305.28 ± 47.81	3481.40 ± 1762.22	3105.37 ± 2081.06
Acetol (Hydroxyacetone)	/	1,149,003.42 ± 1,406,347.70	845,922.62 ± 98,544.09	58,4267.80 ± 658,639.24	199,892.92 ± 8807.73
1-Heptanol	1000.00	59.07 ± 19.30	0.98 ± 1.20	68.37 ± 89.05	417.99 ± 219.96
**Higher Alcohols (Terpenoids)**					
Citronellol	100.00	48.12 ± 15.10	48.71 ± 8.08	54.14 ± 2.00	63.99 ± 26.85
D-Limonene	15.00	4.02 ± 0.33	3.34 ± 0.16	3.42 ± 0.00	3.36 ± 0.03
Nerol	700.00	91.27 ± 2.33	92.94 ± 1.10	95.19 ± 6.15	100.99 ± 5.50
**Fatty Acids**					
Isobutyric acid (2-Methylpropanoic acid)	8100.00	1697.46 ± 769.36	4137.83 ± 1287.62	5486.73 ± 2190.75	2880.92 ± 889.67
Decanoic acid	1000.00	291.31 ± 21.27	236.45 ± 8.57	260.31 ± 30.29	299.53 ± 8.21
Propionic acid	/	5373.85 ± 143.57	5822.57 ± 160.24	6472.68 ± 329.30	6642.6819 ± 837.4474
Benzoic acid	/	1151.38 ± 1.99	1149.27 ± 1.31	1160.83 ± 10.16	1177.61 ± 3.23
**Volatile Phenols**					
4-Ethylphenol	140.00	96.39 ± 0.04	96.34 ± 0.00	96.45 ± 0.02	96.40 ± 0.06
Phenol	/	64.68 ± 1.19	65.46 ± 0.33	67.27 ± 1.01	67.96 ± 2.70
**Other Compounds**					
Furfural	14,100.00	99.15 ± 31.39	24.98 ± 29.05	118.95 ± 47.83	77.11 ± 49.80
trans-β-Ionone	/	7.48 ± 0.02	7.52 ± 0.02	7.49 ± 0.07	7.51 ± 0.03
cis-4-Methyl-5-butyldihydro-2(3H)-furanone (cis-Whiskey lactone)	/	275.00 ± 0.10	275.91 ± 0.18	276.23 ± 0.06	275.17 ± 0.25

Note: “/” indicates odor threshold not available. Threshold values sourced from the relevant literature [26,28].

## Data Availability

The original contributions presented in this study are included in the article/Supplementary Material. Further inquiries can be directed to the corresponding author.

## Supplementary Materials

The following supporting information can be downloaded at: https://www.mdpi.com/article/10.3390/foods15152652/s1. Table S1. Odor activity values (OAV) of aroma compounds in Fu tea–Marselan wine. Figure S1. Permutation test results for the OPLS-DA model. Note: Red points represent R^2^Y values of the actual model, and blue points represent Q^2^ values after random permutation. All blue points lie below the red points, and the regression line slopes downward to the left, indicating no model overfitting and confirming result reliability. Figure S2. VIP heatmap of monomeric phenolic compounds. Note: VIP values > 1 are considered indicative of key differential metabolites. Figure S3. Mfuzz clustering analysis of key differential monomeric phenols. Note: Subclasses represent clusters of metabolites exhibiting similar variation trends. Figure S4. Volcano plots depicting differential OAV values of aroma compounds in tea wine. (a) Multi-group differential volcano plot. Y-axis: magnitude of Log_2_FC; values > 0 indicate upregulation, values < 0 indicate downregulation. Point color: green indicates statistical significance (*p* < 0.05), orange indicates non-significant. Labeled points represent the top N compounds by |Log_2_FC| for each comparison. (b) Multi-group differential volcano plot stratified by up-/downregulation. Y-axis: magnitude of Log_2_FC. Point color: blue indicates upregulated, purple indicates downregulated, orange indicates non-significant. Bar plot numbers indicate counts of up- and downregulated compounds for each comparison. Figure S5. K-means clustering analysis of aroma-active compounds (OAV > 1). Note: *Y*-axis: standardized compound content. *X*-axis: sample groups. Subclass numbers denote clusters of compounds exhibiting similar variation trends. Figure S6. Correlation heatmap between volatile compounds and monomeric phenolic substances. Note: Upper right triangle: correlation heatmap among monomeric phenolic compounds. Cell color indicates correlation coefficient magnitude; red denotes positive correlation, blue denotes negative correlation. Numerical values within cells represent correlation coefficients, and asterisks indicate statistical significance: *** *p* < 0.01, ** *p* < 0.01, * *p* < 0.05. Lower left connections represent Mantel test results between OAV of aroma compounds and monomeric phenols. Line thickness indicates overall correlation coefficient magnitude; line color denotes statistical significance of correlation (red: positive; blue: negative). Figure S7. Morphology of Fu brick tea with characteristic “golden flowers” (*Eurotium cristatum* colonies) on the tea surface. (a) Intact tea brick; (b) close-up view of the tea surface showing the fungal colonies.

## Abbreviations

The following abbreviations are used in this manuscript:

ANOVA	Analysis of variance
C*	Chroma
CIELab	Commission Internationale de l’Éclairage Lab color space
E. cristatum	Eurotium cristatum
GC-MS	Gas chromatography-mass spectrometry
h°	Hue angle
HPLC	High-performance liquid chromatography
HS-SPME	Headspace solid-phase microextraction
L*	Lightness
LOQ	Limit of quantification
OAV	Odor activity value
OPLS-DA	Orthogonal partial least squares discriminant analysis
OT	Odor threshold
PCA	Principal component analysis
S. cerevisiae	Saccharomyces cerevisiae
SD	Standard deviation
VIP	Variable importance in projection
