# Supplementary material for: Synergistic Enhancement of Phenolic Profile, Color Stability, and Aroma Complexity in Marselan Wine Through Co-Fermentation with Fu Tea
Source: Foods. 2026 Jul 28;15(15):2652. doi: 10.3390/foods15152652 (PMC13465712; doi:10.3390/foods15152652)
Supplement: Supplementary file 1 [file foods-15-02652-s001.zip › foods-4421448-supplementary.pdf]

Synergistic Enhancement of Phenolic Profile, Color Stability, and Aroma Complexity in Marselan Wine through Co-Fermentation with Fu Tea

Authors: Dolkar, Bai H.X., Wang Z.Y., Yang J.H.

Table S1. Odor activity values (OAV) of aroma compounds in Fu tea–Marselan wine.

Aroma compounds	*OT[36,37]（μg/L）*	*OAV（0g/L（Control）*	*OAV (0.5g/L)*	*OAV (1.0g/L)*	*OAV (1.5g/L)*
Ethyl acetate	7500.00	9.68±5.156	5.527±0.149	6.163±0.48	7.573±2.545
Ethyl butyrate	20.00	8.768±2.945	6.359±0.686	7.141±0.496	8.386±3.236
Isoamyl alcohol (3-Methylbutanol)	30000.00	8.153±2.754	6.269±3.051	10.027±0.236	6.475±3.977
4-Ethylguaiacol	22.00	6.869±0	6.869±0	6.869±0	6.87±0.001
α-Terpineol	6.00	6.542±2.404	1.043±0.01	1.054±0.037	3.789±0.99
Ethyl hexanoate	14.00	33.757±13.083	7.579±0.542	16.095±5.364	7.072±8.175
Ethyl octanoate	874.00	3.395±1.197	2.597±0.397	3.68±0.221	2.372±0.831
3-(Methylthio)-1-propanol (Methionol)	1000.00	2.176±0.133	1.909±0.594	2.27±0.615	2.224±0.446
Ethyl 2-methylbutyrate	18.00	12.531±2.47	4.997±1.824	7.341±6.464	3.203±0.49
Isoamyl acetate	30.00	11.424±4.871	9.024±0.385	10.03±2.025	11.934±3.382
Isobutanol (2-Methylpropanol)	40000.00	1.636±0.449	1.212±0.272	1.513±0.33	1.469±1.007
Hexyl isovalerate	3.00	1.618±0.369	1.814±0.418	1.809±0.084	1.678±0.393
Octanoic acid	500.00	1.526±0.038	1.45±0.043	1.487±0.037	1.503±0.077
Ethyl decanoate	200.00	1.475±0.44	0.894±0.433	0.735±0.008	0.893±0.313
Ethyl 3-methylbutyrate (Ethyl isovalerate)	3.00	1.46±1.102	1.686±0.043	3.307±0.708	3.141±1.22
1-Propanol	50000.00	1.468±0.097	1.206±0.254	1.122±0.444	1.192±0.404
Phenylethanol (2-Phenylethanol)	14000.00	1.375±0.492	2.556±0.377	2.557±0.653	1.774±0.607
4-Methylpentanol (Isohexanol)	500000.00	0.000±0	0.000±0	0.000±0	0.000±0
Hexyl acetate	670.00	0.000±0	0.000±0	0.000±0	0.001±0
4-Ethylphenol	140.00	0.689±0	0.688±0	0.689±0	0.689±0
Acetic acid	200000.00	0.561±0.11	0.727±0.054	1.454±0.395	1.879±0.991
Citronellol	100.00	0.481±0.151	0.487±0.081	0.541±0.02	0.64±0.268

Decanoic acid	1000.00	0.291±0.021	0.236±0.009	0.26±0.03	0.3±0.008
D-Limonene	15.00	0.268±0.022	0.223±0.01	0.228±0	0.224±0.002
Isobutyric acid (2-Methylpropanoic acid)	8100.00	0.21±0.095	0.511±0.159	0.677±0.27	0.356±0.11
Amyl acetate (Pentyl acetate)	30.00	0.209±0.057	0.189±0.035	0.218±0.003	0.174±0.08
Phenethyl acetate	73.00	0.193±0.044	0.263±0.02	0.273±0.004	0.237±0.059
Nerol	700.00	0.13±0.003	0.133±0.002	0.136±0.009	0.144±0.008
Diethyl succinate	6000.00	0.087±0.032	0.163±0.003	0.241±0.008	0.14±0.048
Linalool	200.00	0.05±0.017	0.584±0.128	0.978±0.48	0.819±0.113
1-Pentanol	64000.00	0.05±0.012	0.074±0.003	0.086±0.002	0.06±0.023
1-Heptanol	1000.00	0.059±0.019	0.001±0.001	0.068±0.089	0.418±0.22
Ethyl laurate	1500.00	0.042±0.014	0.024±0.01	0.023±0.003	0.027±0.011
Furfural	14100.00	0.007±0.002	0.002±0.002	0.008±0.003	0.006±0.004
Ethyl nonanoate	1300.00	0.007±0.001	0.014±0.002	0.008±0	0.014±0.002
Benzyl alcohol	200000.00	0.004±0	0.005±0	0.005±0	0.005±0
Isooctanol (2-Ethylhexanol)	8000.00	0.002±0.001	0.009±0.001	0.013±0	0.002±0.001
3-Methylpentanol	15000.00	0.001±0.001	0.003±0	0.002±0.001	0.002±0.001
Propionic acid	/	/	/	/	/
trans-β-Ionone	/	/	/	/	/
Benzoic acid	/	/	/	/	/
Phenol	/	/	/	/	/
Farnesol	/	/	/	/	/
Acetol (Hydroxyacetone)	/	/	/	/	/
2-Hexen-1-ol	/	/	/	/	/
Ethyl salicylate	/	/	/	/	/
cis-4-Methyl-5-butyldihydro-2(3H)-furanone (cis-Whiskey lactone)	/	/	/	/	/
Methyl salicylate	/	/	/	/	/

Note: OAV > 1 indicates significant contribution to overall aroma. Only compounds with OAV > 1 in at least one treatment group are displayed.

Synergistic Enhancement of Phenolic Profile, Color Stability, and Aroma Complexity in Marselan Wine through Co-Fermentation with Fu Tea
Authors: Dolkar, Bai H.X., Wang Z.Y., Yang J.H.

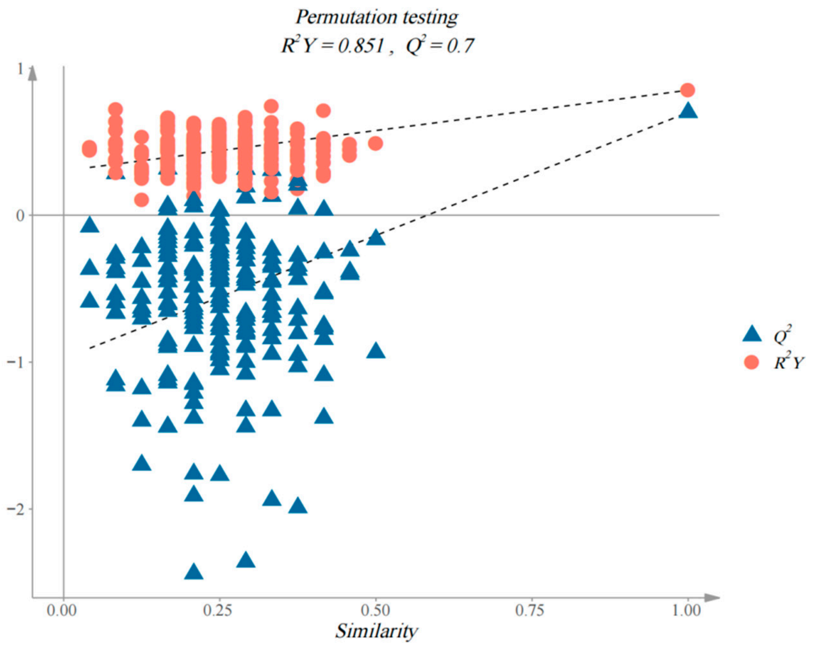

Figure S1. Permutation test results for the OPLS-DA model. Note: Red points represent R^2Y values of the actual model, and blue points represent Q^2 values after random permutation. All blue points lie below the red points, and the regression line slopes downward to the left, indicating no model overfitting and confirming result reliability.

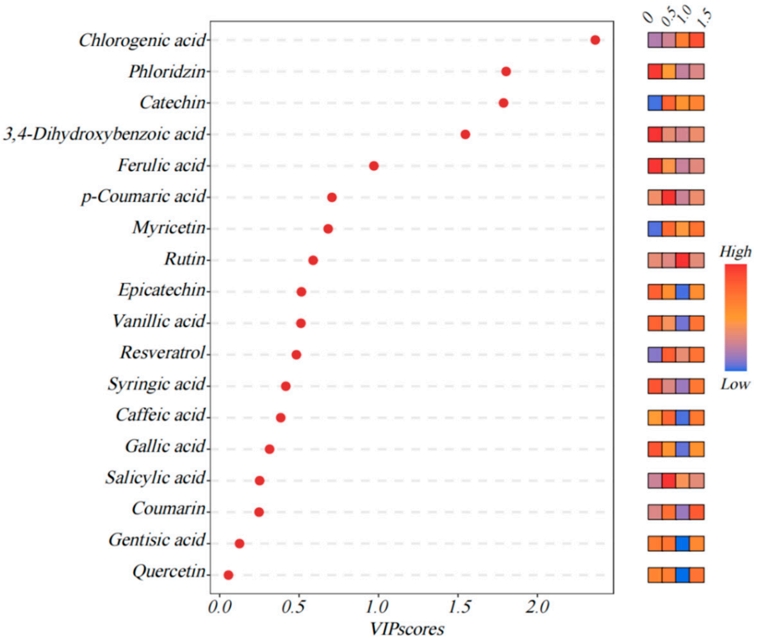

Figure S2. VIP heatmap of monomeric phenolic compounds. Note: VIP values > 1 are considered indicative of key differential metabolites.

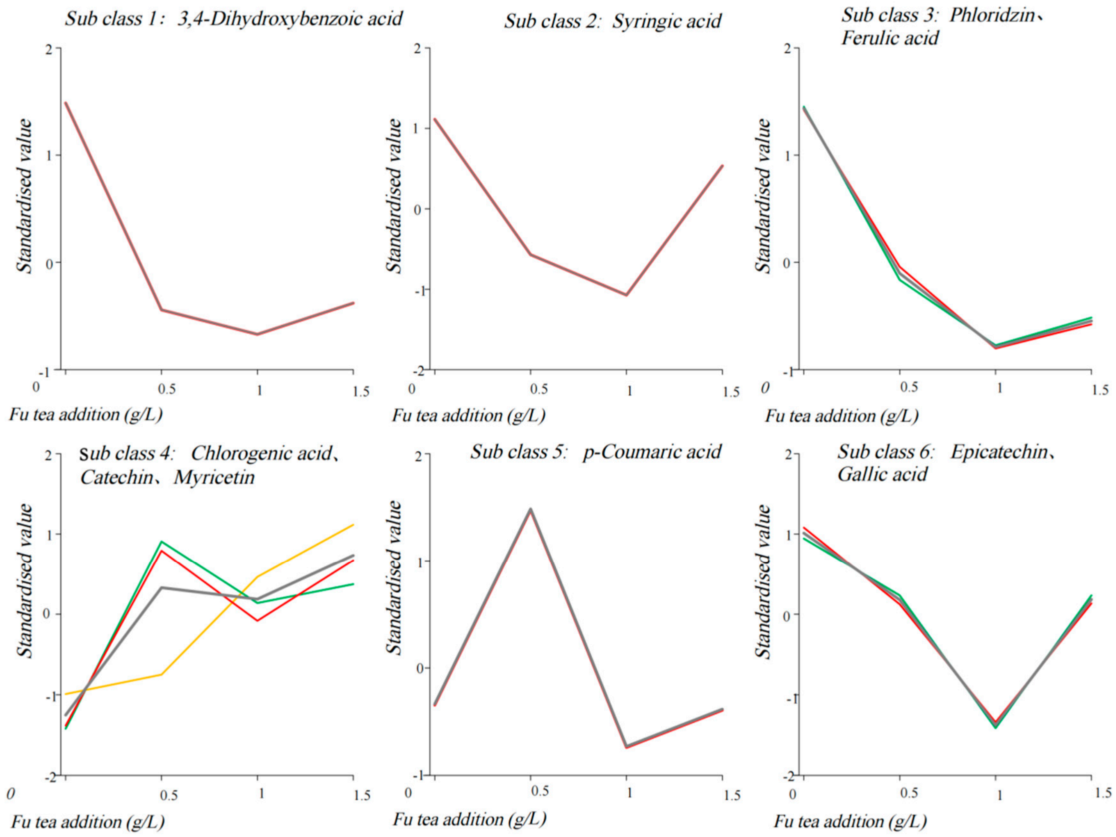

Figure S3. Mfuzz clustering analysis of key differential monomeric phenols. Note: Subclasses represent clusters of metabolites exhibiting similar variation trends.

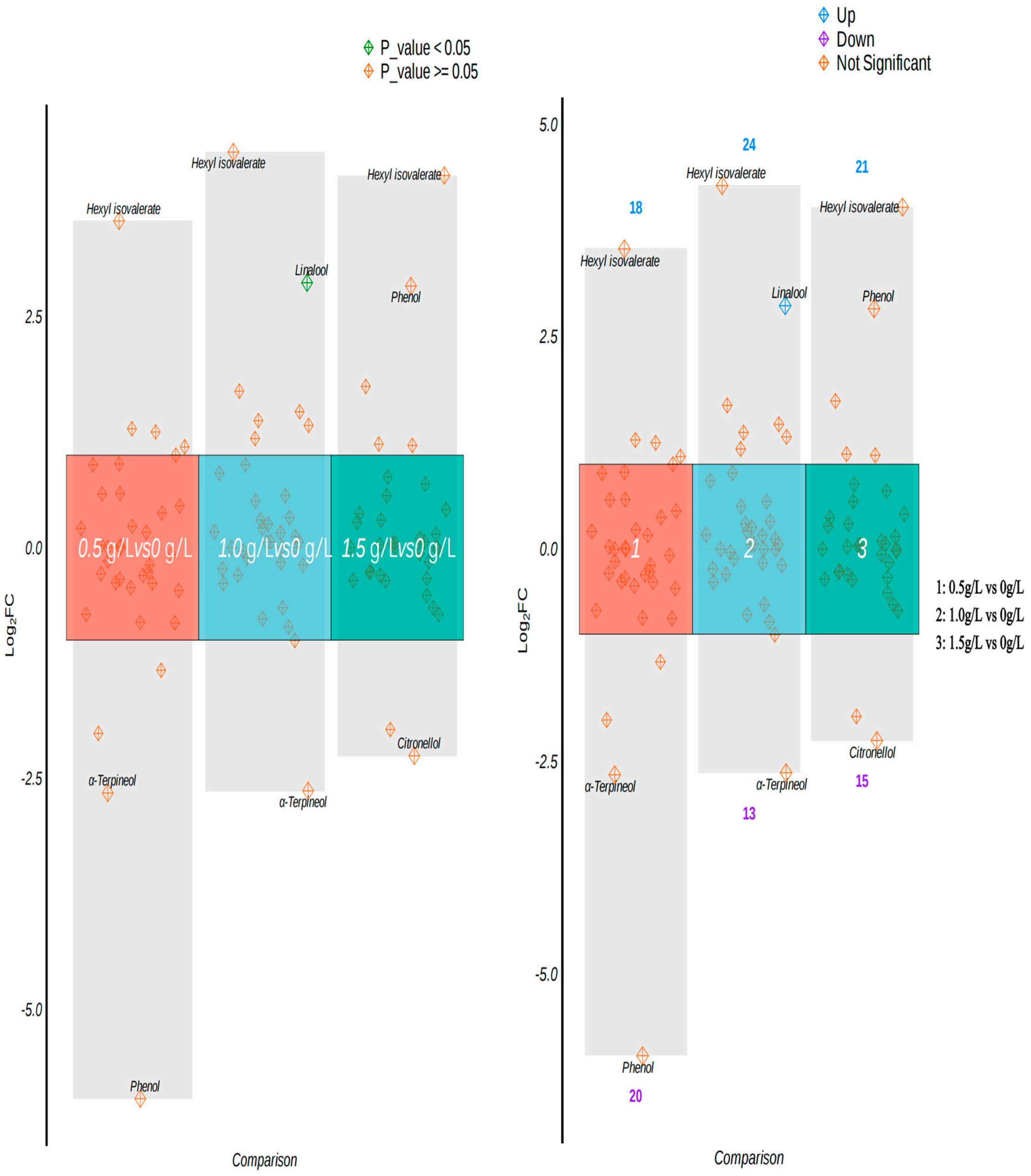

(**a**) (**b**)

Figure S4. Volcano plots depicting differential OAV values of aroma compounds in tea wine. (a) Multi-group differential volcano plot. Y-axis: magnitude of Log_2FC; values > 0 indicate upregulation, values < 0 indicate downregulation. Point color: green indicates statistical significance ($p < 0.05$), orange indicates non-significant.

Labeled points represent the top N compounds by |Log$_2$FC| for each comparison. (b) Multi-group differential volcano plot stratified by up-/downregulation. Y-axis: magnitude of Log$_2$FC. Point color: blue indicates upregulated, purple indicates downregulated, orange indicates non-significant. Bar plot numbers indicate counts of up- and downregulated compounds for each comparison.

Synergistic Enhancement of Phenolic Profile, Color Stability, and Aroma Complexity in Marselan Wine through Co-Fermentation with Fu Tea Authors: Dolkar, Bai H.X., Wang Z.Y., Yang J.H.

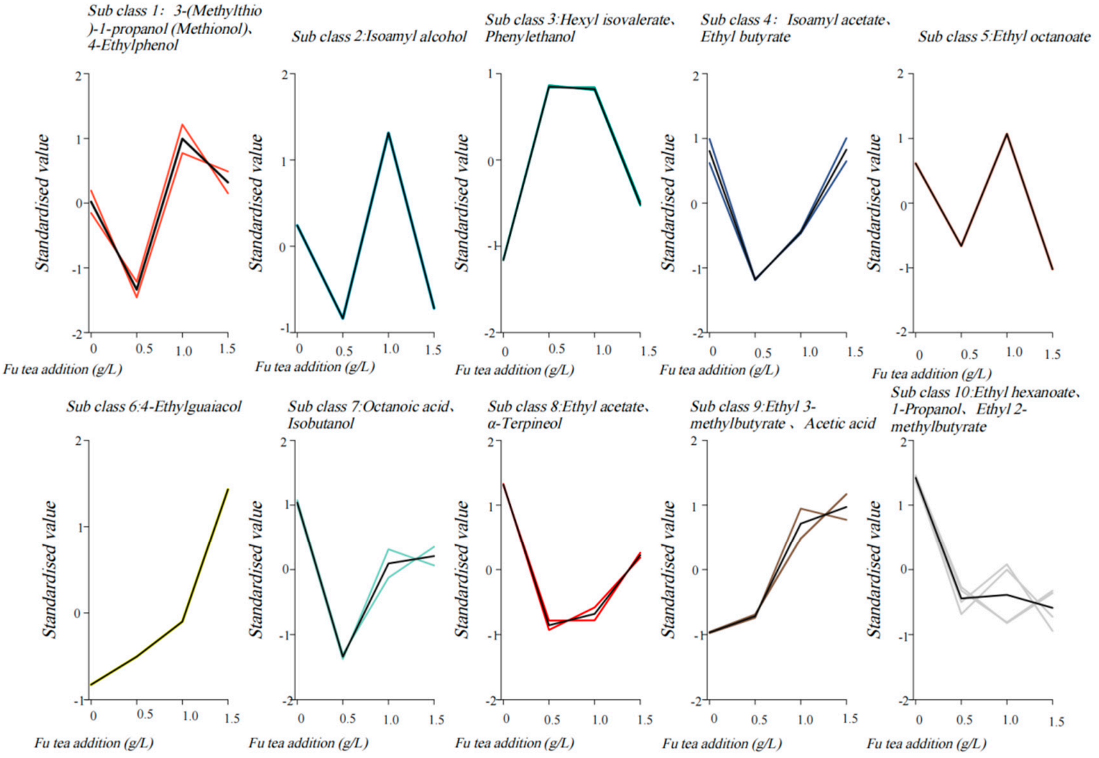

Figure S5. K-means clustering analysis of aroma-active compounds (OAV > 1). Note: Y-axis: standardized compound content. X-axis: sample groups. Subclass numbers denote clusters of compounds exhibiting similar variation trends.

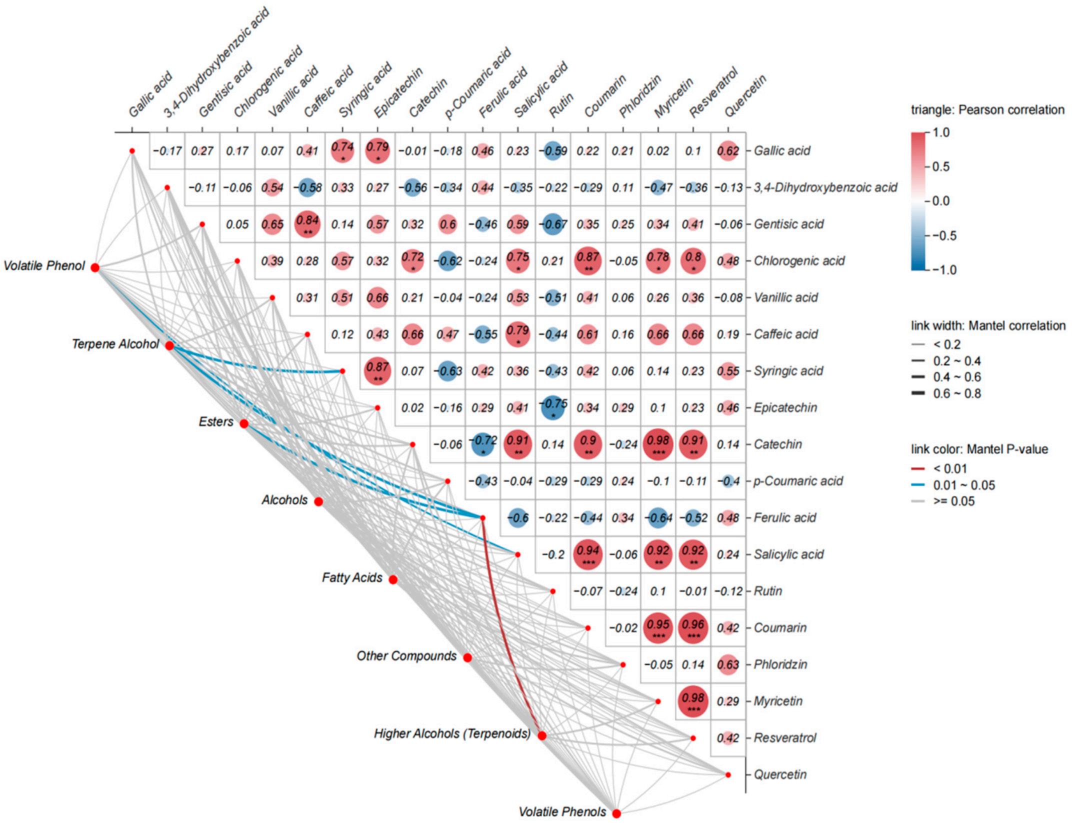

Figure S6. Correlation heatmap between volatile compounds and monomeric phenolic substances. *Note: Upper right triangle: correlation heatmap among monomeric phenolic compounds. Cell color indicates correlation coefficient magnitude; red denotes positive correlation, blue denotes negative correlation. Numerical values within cells represent correlation coefficients, and asterisks indicate statistical significance: *** $p < 0.01$, ** $p < 0.01$, * $p < 0.05$. Lower left connections represent Mantel test results between OAV of aroma compounds and monomeric phenols. Line thickness indicates overall correlation coefficient magnitude; line color denotes statistical significance of correlation (red: positive; blue: negative).*

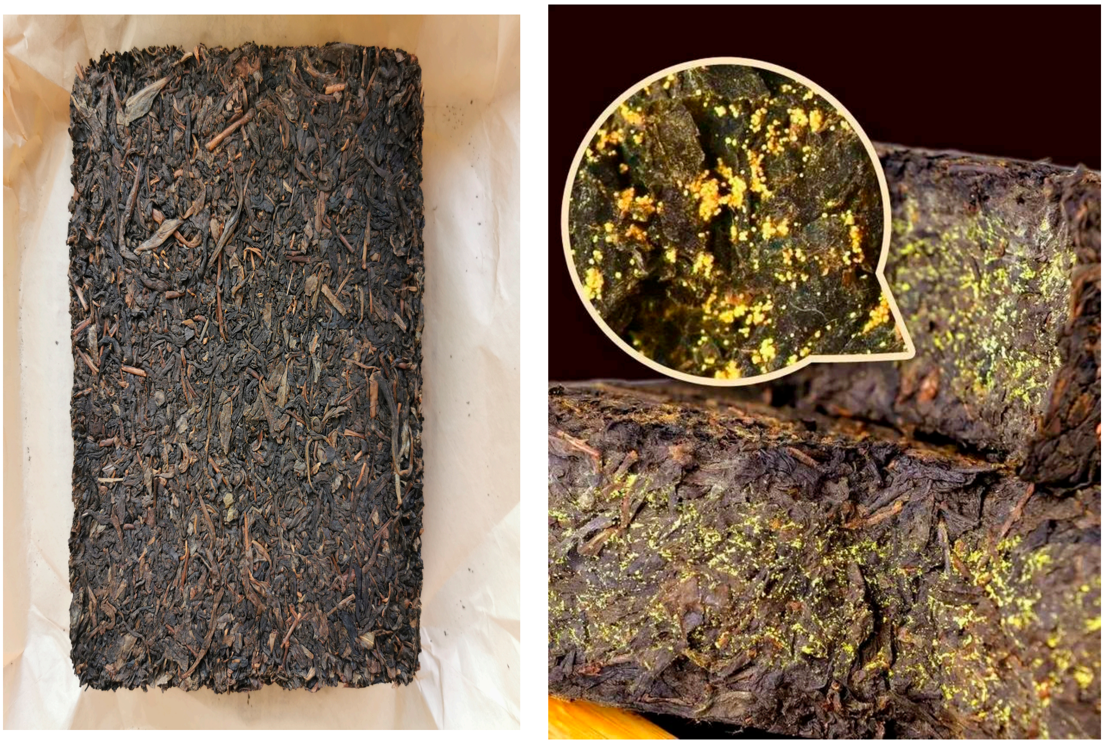

(**a**) (**b**)

Figure S7. Morphology of Fu brick tea with characteristic "golden flowers" (*Eurotium cristatum* colonies) on the tea surface. (a) Intact tea brick; (b) close-up view of the tea surface showing the fungal colonies.
